# Preparation of DOPC and DPPC Supported Planar Lipid Bilayers for Atomic Force Microscopy and Atomic Force Spectroscopy

**DOI:** 10.3390/ijms14023514

**Published:** 2013-02-06

**Authors:** Simon J. Attwood, Youngjik Choi, Zoya Leonenko

**Affiliations:** 1 Department of Physics and Astronomy, University of Waterloo, Waterloo, ON N2L 3G1, Canada; E-Mail: simonjamesattwood@gmail.com; 2 Department of Biology, University of Waterloo, Waterloo, ON N2L 3G1, Canada; E-Mail: y.vince.choi@gmail.com; 3 Waterloo Institute for Nanotechnology, University of Waterloo, Waterloo, ON N2L 3G1, Canada

**Keywords:** DOPC, DPPC, AFM, force spectroscopy, supported lipid bilayer, vesicle fusion, breakthrough forces, force volume

## Abstract

Cell membranes are typically very complex, consisting of a multitude of different lipids and proteins. Supported lipid bilayers are widely used as model systems to study biological membranes. Atomic force microscopy and force spectroscopy techniques are nanoscale methods that are successfully used to study supported lipid bilayers. These methods, especially force spectroscopy, require the reliable preparation of supported lipid bilayers with extended coverage. The unreliability and a lack of a complete understanding of the vesicle fusion process though have held back progress in this promising field. We document here robust protocols for the formation of fluid phase DOPC and gel phase DPPC bilayers on mica. Insights into the most crucial experimental parameters and a comparison between DOPC and DPPC preparation are presented. Finally, we demonstrate force spectroscopy measurements on DOPC surfaces and measure rupture forces and bilayer depths that agree well with X-ray diffraction data. We also believe our approach to decomposing the force-distance curves into depth sub-components provides a more reliable method for characterising the depth of fluid phase lipid bilayers, particularly in comparison with typical image analysis approaches.

## 1. Introduction

The surfaces of cell plasma membranes play a pivotal role in many biological processes including cell recognition, signalling, selective-ion transfer, adhesion and fusion [1]. The composition and lateral organisation of native membranes are complex, consisting for example of mixtures of phospholipids, glycolipids and various proteins. Such complexity makes the task of identifying the specific effects of membrane interactions with other molecules very difficult. Therapeutic drugs or protein molecules may target specific receptors, but also may interact non-specifically with the lipid membrane itself [2,3]. By simplifying the system, it is possible to systematically study the sub-components of cellular membranes and therefore gain valuable insights that would otherwise be obscured.

Atomic Force Microscopy is a very powerful technique that can be used to study not only the topographical changes but also a range of biomechanical properties. There has been a lot of interest recently in planar supported lipid bilayers (SLB) as model systems, comprised of either single or multiple component lipids, prepared either using vesicle fusion [4–12], or Langmuir–Blodgett or Langmuir–Schaefer deposition [13–15]. There are generally two approaches to studying these systems with the Atomic ForceMicroscope. Firstly, AFM imaging can be performed by scanning the AFMprobe across the surface of a lipid bilayer, which provides information on the topographical characteristics of the supported lipid bilayer, such as the lateral extent of domains, roughness and height of patches relative to the substrate. Then, after addition of an effector molecule of interest, the surface topography can be re-assessed. We can also find the timescale of the interaction by imaging the surface after incremental time steps and at each point assess the changes. Examples of these type of studies include lipid interactions with anesthetic halothane [16,17], ethanol [16], antibiotic azithromycin [18,19], immunodeficiency peptide [7], peptide gramicidin [20], amyloid beta [21–24], model peptide WALP23 [25].

The second approach is to apply force spectroscopy to assess the biomechanical changes due to some effector molecule. In this technique AFM probes are brought towards the supported lipid bilayer and a load increasingly applied until the bilayer ruptures and the probe senses the underlying hard substrate. Afterwards the probe is withdrawn and the cycle is repeated many times. The rupture events are manifested by a well-defined discontinuity in the force-distance approach curves, which can subsequently be analysed to determine the magnitude of the rupture force or break-through-force. The average or most probable rupture force has shown to be a fingerprint for the intrinsic properties of the bilayer. The effect of pH [26], ionic strength of medium [27], deposition pressure [28], temperature [29] and head/tail group composition [30] on membrane structure and function have all been studied. Furthermore, the effect of various proteins and drugs have also been studied, including Myelin based protein [31,32], cytochrome-c [31], bax protein [33], cholesterol [34–36], Synapsin I [37], general anesthetic halothane [17] and antibiotic azithromycin [19]. Atomic force spectroscopy (AFS) is commonly performed in a force volume mode in order to collect a statistically sound set of data. This requires defect-free supported planar lipid bilayers covering extended areas. In all of these studies an essential prerequisite is a well-developed protocol that can consistently be used to prepare lipid bilayers [38].

In the current work we detail two protocols that have been developed for preparing both fluid and gel phase planar bilayers on mica for use in AFM studies. Dioleoylphosphatidylcholine (DOPC) has a transition temperature of–16.5 °C [39] and therefore exists in the fluid like liquid crystalline state (*L_β_*) at room temperature. Dipalmitoylphosphatidylcholine (DPPC) has a transition temperature of 41.3 °C [40] and therefore exists in the solid-like gel state (*L_β_*) at room temperature. Lipids in different states can affect membrane functionality very differently and we therefore chose two lipids to represent the two main lipid phase classes. Furthermore, phospholipids containing the choline group moiety are the most abundant class in eukaryotic cells [41]. We also note that DOPC and DPPC are two of the most common model lipid systems studied.

We report defect-free bilayers that are ideally suited for AFM studies. We highlight the most significant experimental parameters, and introduce tests that can be used to confirm the presence of bilayer. We present optimization approaches to account for solution to solution differences that are difficult to control. We then highlight the major differences between fluid and gel phase lipids by presenting a protocol for the formation of DPPC bilayers. We illustrate through experiment the effect of parameters such as solution temperature, cooling rate, incubation time, concentration and ionic strength.

Without a thorough understanding of bilayer preparation, it is easy to produce misleading results. Even mature protocols are reported to take several months of practice before these model systems can be accurately and reliably reproduced [38]. We hope to highlight the relative importance of various experimental parameters, illustrate the significant differences between gel and fluid phase lipids, and present important tests for bilayer assessment. Debate still continues about the best way to prepare bilayer samples and the important experimental parameters as evident by every single laboratory using a different protocol. We hope that through this work, scientists new to the field can quickly and reliably produce model bilayer systems for their study.

Finally, we illustrate the effectiveness of the DOPC protocol by assessing the DOPC surface with force spectroscopy. Without a reliable, robust and defect-free bilayer surface, it is easy for the tip to get contaminated, producing inconsistent and misleading results. We demonstrate that rupture force and depth values can easily be obtained in good agreement with other AFM studies. We believe that obtaining the depth characteristics of a bilayer from the force spectroscopy measurements is a much more accurate and representative measure of the bilayer thickness. Our values agree well with previous X-ray diffraction studies.

## 2. Results and Discussion

### 2.1. DOPC Bilayers from Vesicles in Water

The AFM is a very powerful tool for determining the topography of materials at the nanoscale and has the distinct advantage over other techniques in that it can be used in liquid over a range of salt concentrations and pH values. This makes it particularly amenable to the study of biological systems where physiological conditions are important. In the case of synthetic bilayers, which are important cell analogues, experiments must be carried out at least in pure water, as their assembly and stability is primarily driven by the hydrophobicity of these amphiphilic molecules.

A deficiency of the AFM is its inability to truly probe three dimensions. Topography maps lack a depth component into the sample and are really two dimensional surface maps. From a practical viewpoint, it is therefore very difficult to distinguish single bilayers that completely cover the surface from multilayers and/or bare mica, all of which would produce a completely featureless image. There are tricks however that can be employed to overcome some of these problems.

**Figure 1:**
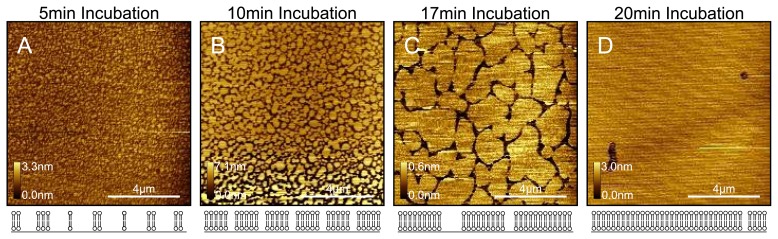
Time series of DOPC bilayer formation. Four separate samples of DOPC bilayers on mica were prepared that were incubated for (**A**) 5min; (**B**) 10min; (**C**) 17min and (**D**) 20min. Below each image is an illustration of the state of the lipid coverage across the mica surface. The time series experiment is a good way of determining that a complete single bilayer covers the mica surface. Without doing the time series experiment, it is very difficult to distinguish a complete single bilayer from multilayer or even bare mica. In addition, when faced with a DOPC sample with partial patchy coverage, it is a simple task to prepare a new sample with a slightly increased incubation time that will result in continuous coverage. All images were taken in pure water at room temperature.

Protocols for the preparation of DOPC liposome solutions and sample preparation by vesicle fusion are described in detail in the experimental section. In order to produce and verify that a complete bilayer has been formed on the mica surface, we find it necessary to perform a time series of experiments. That is, we prepare several samples each incubated with the liposome solution for slightly different times. Thus, we are able to capture the sample at different stages during formation and, by washing, halt any further progress. As shown in Figure 1, this allows us to very precisely determine the exact experimental conditions for the specific liposome solution being used, which will produce complete coverage (we observe continuous bilayers for *>* 30 *μ*m^2^ areas). There are several methods for confirming whether we observe single bilayer patches spreading across a mica surface versus patches of bilayer forming on top of a complete first bilayer (or multilayer). Firstly, the phase signal, which is thought to reflect differences in mechanoelastic and surface chemical properties, can be used; if we see a large difference in phase (1–2 degrees for Agilent AFM) between the bilayer patches and the underlying surface, then this is a good indication that we are observing mica beneath the bilayer patches. However, if the tip gets contaminated, which is very likely whilst imaging the soft DOPC bilayers, then the contrast will not be as great and making a firm conclusion is difficult. We also know that if we incrementally decrease the time or concentration any further than as in the least covered surface, we will only see featureless samples, indicative of bare mica.

It should be noted that it has previously been thought that the addition of calcium or other divalent cations was an essential step to forming bilayers on mica surfaces [38,42–44]. However we have unequivocally demonstrated here that bilayers on mica can be formed by vesicle fusion in pure water. Such systems may be useful for example when trying to exclude ion mediated interactions.

**Figure 2:**
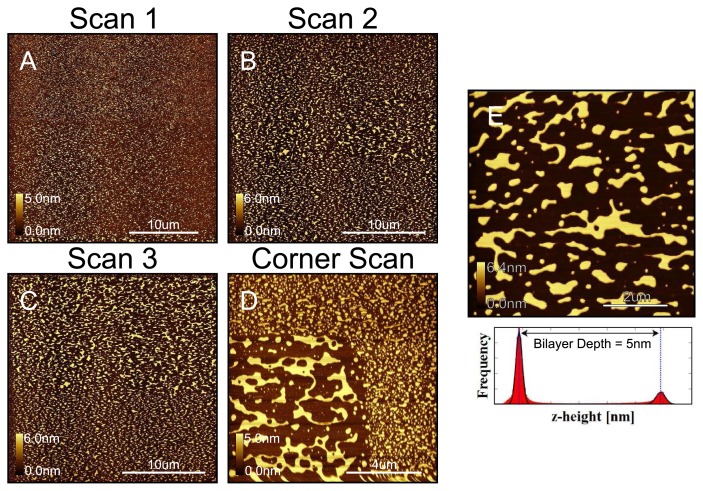
Tip induced DOPC gathering effects on sparsely covered samples indicating both the fluidity of DOPC and the weak attraction of DOPC with mica. (**A**) DOPC sample prepared with a very sparse coverage of DOPC; (**B**) and (**C**) successive scans of sample in A cause the lipid to be drawn together, making larger patches; (**D**) The effect is most clearly seen when a corner at the boundary between a previously scanned region, and an untouched region is scanned; (**E**) The distribution of pixel heights shows two distinct populations, representing the mica surface and the surface of the bilayer. The bilayer depth is found by subtracting the Gaussian fitted peaks of the two populations. The value of 5 nm obtained here agrees well with expected values.

In Figure 2 we illustrate the delicate, fluidic nature of DOPC. We first prepared a sparsely covered sample of DOPC on mica, and then scanned successively over the same area. As can be seen, the tip drags the lipid together, forming successively larger patches, with the space between them growing. The effect is most obvious when the boundary between a previously scanned area and an untouched area is observed, such as at the corner. By carefully flattening an image of such patches, we are able to observe two distinct populations, representing the mica surface and the top surface of the bilayer. By fitting two Gaussian distributions and then subtracting the difference between the peak heights, we find that the bilayer depth is 5 nm. The precision of this measurement is very high (standard error of the mean < 0.02%), however the largest uncertainty in this measurement is due to the force setpoint. By scanning with a higher force, it is easy to compress the bilayer, measuring slightly less than the “true” depth, and by scanning with a low force, it is possible that longer range repulsive forces play a role, resulting in an overestimation. We believe a quantitative approach to depth measurement of soft bilayer systems can only be achieved by analysing force distance curves.

**Figure 3:**
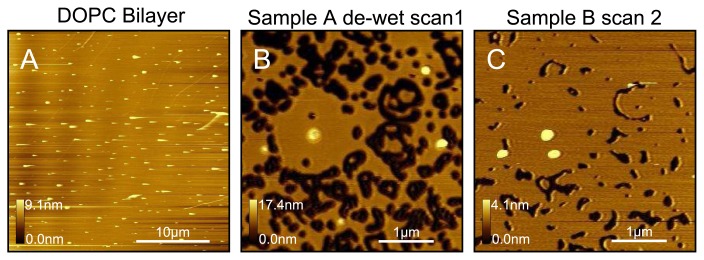
Illustration of the dewetting test used to confirm bilayer coverage. (**A**) A sample is prepared using DOPC liposomes. Apart from some protrusions the sample is defect-free, making a confirmation of the state of the sample (mica, single bilayer, multilayer) difficult; (**B**) Sample instantaneously dewetted and then rehydrate. The holes confirm the presence of a single bilayer; (**C**) After a short period the holes begin to close, demonstrating again the fluidic nature of DOPC. All images were taken in pure water at room temperature.

Another useful technique for testing a bilayer surface is the de-wetting approach. In Figure 3A we observe a predominantly flat sample, except for some protrusions. It is difficult from this single image to be certain as to whether we are observing a sample predominantly consisting of uncoated mica but with a few vesicles adsorbed to the surface, or whether there is a complete bilayer covering the mica but with a few vesicles either adsorbed or existing in a trapped state [6,43,45]. By simply removing some of the water from the fluid cell so that the surface de-wets for a few seconds and then replacing the water quickly, we observe many holes as shown in Figure 3B. This clearly demonstrates that the original surface was in fact a complete bilayer. Again the fluidity of this DOPC sample is demonstrated when we scan some time later, over exactly the same area, as in Figure 3C, where we observe the holes to have decreased in size. In contrast to the de-wetting technique, we observe that increasing the strength of the washing stream or the volume used to wash the sample has no perceivable effect.

### 2.2. DPPC Bilayers from Vesicles in Water

Whereas liposomes of DOPC in solution have a transition temperature of −16.5 °C [39], liposomes of DPPC have been determined to have a transition temperature of 41.3 °C [40]. This means that DOPC is in the fluid phase at room temperature, whereas DPPC is in the gel phase. When bilayers are attached to a mica surface, the proximal and distal leaflets are decoupled due to the strong interaction of the proximal leaflet with the mica surface [46] leading to a broadening of the transition temperature to between 41 °C and 46 °C. AFM based studies demonstrated that there is a transition temperature width of 10 °C [47] for single bilayers on mica. Even with substrate induced broadening of the transition temperature, it is clear that DPPC is in the gel phase at room temperature. The dynamics of vesicle fusion are completely different for lipids in the gel phase compared with the fluid phase. Typically it is reported that DPPC vesicles should be heated above the transition temperature (50–60 °C) in order for them to fuse with the mica surface and form planar bilayers [23,38,48]. We typically observe that DPPC vesicles in water deposited at room temperature either partially fuse, or only form a vesicle layer. However, we demonstrate later that for high concentrations of DPPC solution deposited for short time periods before washing, the vesicles will fuse to mica at room temperature.

**Figure 4:**
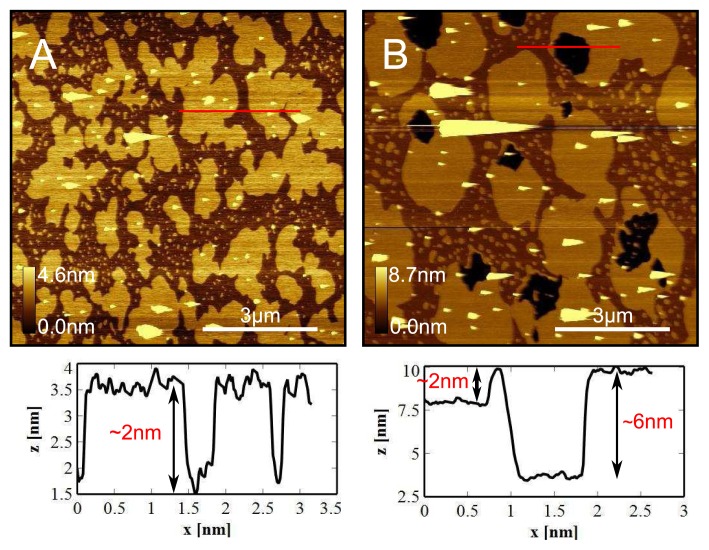
DPPC domains observed at room temperature. (**A**) DPPC liposome solution in pure water was added to a fluid cell at 60 °C. After 5min incubation the sample was washed and allowed to cool with the heater off ~ 5 °Cmin^−1^ . Lipid completely covers the surface with two different domains with a height difference of ~ 2 nm; (**B**) The same sample was reheated to 60 °C, held at that temperature for 5min and cooled at 5 °Cmin^−1^ . Holes in the lipid exposing mica appear after re-heating, which are thought to be due to lipid loss into the liquid.

We prepared DPPC liposomes using the general protocol described in the experimental section, with the lipid at 0.3mgml^−1^. The solution was then added to a fluid cell containing mica maintained at 60 °C using the heating stage. After 5min the sample was washed with water and allowed to cool at room temperature (~ 5 °Cmin^−1^ ). Previous experiments (data not shown) with low lipid concentrations (< 0.5mgml^−1^ ) demonstrated that the vesicles would not fuse to mica at room temperature. However, as shown in Figure 4A, when heating in-situ, the vesicles are able to fuse. We observe complete bilayer coverage across the mica surface, however we very distinctly observe ~ 2 nm high domains. We then reheated the sample to 60 °C, which was maintained for 5min, and then cooled the sample (5 °Cmin^−1^ ) to room temperature. We now observe holes in the bilayer surface exposing mica, due to lipid loss to the water [29], allowing us to clearly see the three levels correlating to bare mica, a low DPPC domain, and a high DPPC domain. The highest domain appears to be about 6 nm in height, consistent with DPPC in its gel phase [25,47,49]. Furthermore, we see that the higher domains have become larger in size and more uniform at their edges, suggesting a re-organization has occurred due to the extra heating step. Similar domains have been reported before [23,29,47,50] at room temperature although the domains were usually much smaller and experiments were carried out in buffer with salt. It is not clear exactly what they are due to, however they are usually attributed to either interdigitation, or tilting of the lipids. These experiments have been repeated using a liposome solution that was prepared straight from powdered DPPC, so as to be completely certain that there are no trace solvents in the solution. The results were qualitatively identical to those presented here.

**Figure 5:**
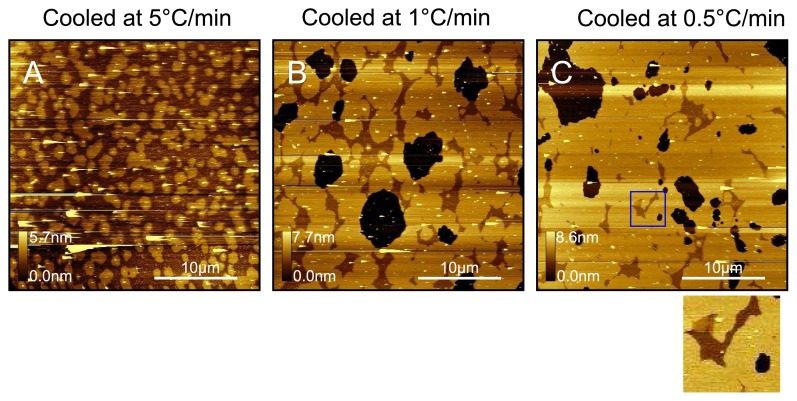
Effect of cooling rate on DPPC bilayers in water. All samples were prepared by incubation with DPPC liposome solution in water at 60 °C and incubation for 5min followed by cooling to room temperature at (**A**) 5 °Cmin^−1^ ; (**B**) 1 °Cmin^−1^ ; (**C**) 0.5 °Cmin^−1^ . The region enclosed by the blue box has been enlarged and is shown below. For this image of DPPC at room temperature, we see three different types of domains with the lowest two being 1.1 nm and 0.6 nm below the upper domain.

As shown in the large area (~ 30 *μ*m^2^ ) scans of Figure 5, we investigated the effect of cooling rate on sample topography. Three separate samples were prepared at cooling rates of 5 °Cmin^−1^, 1 °Cmin^−1^ and 0.5 °Cmin^−1^. There is a clear progression observed in which higher domains cover an increasingly larger proportion of the mica surface relative to the lower domains with decreasing cooling rate. We also observe regions of bare mica in the samples cooled at 1 °Cmin^−1^ and 0.5 °Cmin^−1^, which is a reflection of the increased time spent at higher temperatures as compared with the sample cooled at 5 °Cmin^−1^ . As shown in Figure 5C we sometimes observe three different types of domains, with the lowest two being 1.1 nm and 0.6 nm below the upper domain in this case. It is not clear what the lower domains are due to, however there is likely a decoupling effect due to the proximal leaflet interacting with the mica surface. For all samples we tend also to see some vesicles that are not removed by washing and are likely to be trapped [6,43,45]. However, with the sample cooled at 0.5 °Cmin^−1^ we do see extended regions several micrometers square of defect-free bilayer that could be used to test interactions with other molecules either by surface imaging or force spectroscopy.

**Figure 6:**
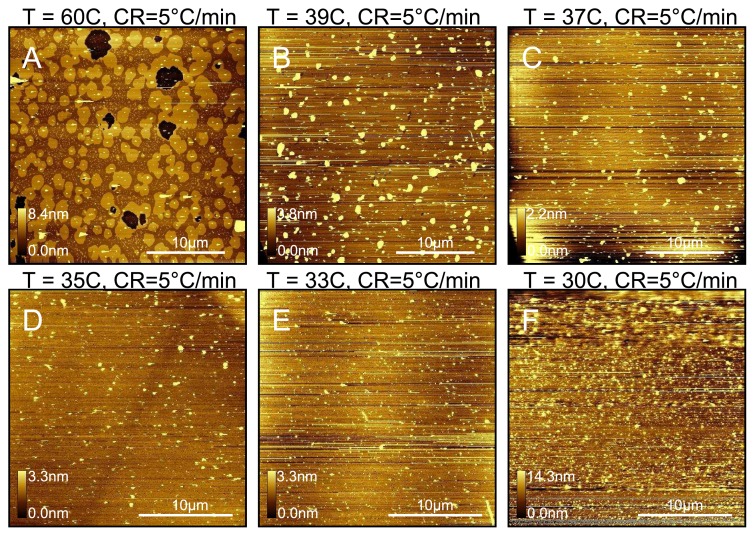
Effect of mica temperature during deposition. All samples were prepared with a cooling rate of 5 °Cmin^−1^ . Mica temperature during deposition was: (**A**) 60 °C; (**B**) 39 °C; (**C**) 37 °C; (**D**) 35 °C; (**E**) 33 °C; (**F**) 30 °C. Lower domains decrease dramatically as a proportion of total lipid coverage between 60 °C and 39 °C, after which a slow decrease is observed to 33 °C. At 30 °C we mostly observe unfused vesicles. Protrusions thought to be trapped vesicles are seen for all samples but dominate for temperatures below 60 °C.

We also tested the effect of changing the temperature of the mica in the fluid cell. When only 39 °C is maintained, we see a dramatic reduction in the number of lower domains compared with a sample prepared at 60 °C as shown in Figure 6. The trend of decreasing lower domains with decreasing temperature continues down to 33 °C where the domains are almost completely eliminated. However, at 30 °C we start to observe unfused vesicles. This suggests that although the transition temperature for a DPPC liposome solution is 41 °C, they will fuse with mica in pure water at and above 33 °C. Thus it seems the minimum temperature for complete vesicle fusion is well below the lipid transition temperature. However, for these samples prepared between 33 °C and 39 °C, we observe many more protrusions across the surface, suggesting that trapped vesicles are more likely to form at these lower temperatures. Reimhult *et al*. [45] made the same observation with eggPC (transition temperature ~ −15 °C) using the Quart Crystal Microbalance technique. We also tested a sample prepared at 33 °C and cooled at 0.5 °Cmin^−1^, for which we observe the lower domains completely eliminated (data not shown as very similar to Figure 6E).

**Figure 7:**
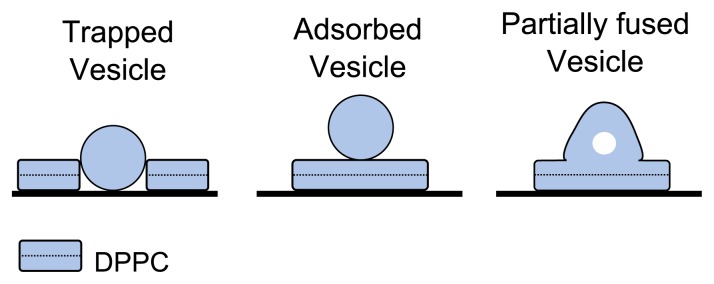
Assigning identity to protrusions observed in bilayer samples. The schematics illustrate three possible variants that may lead to the protrusions seen in DPPC bilayer samples: trapped vesicle, adsorbed vesicle and partially fused vesicle.

**Figure 8:**
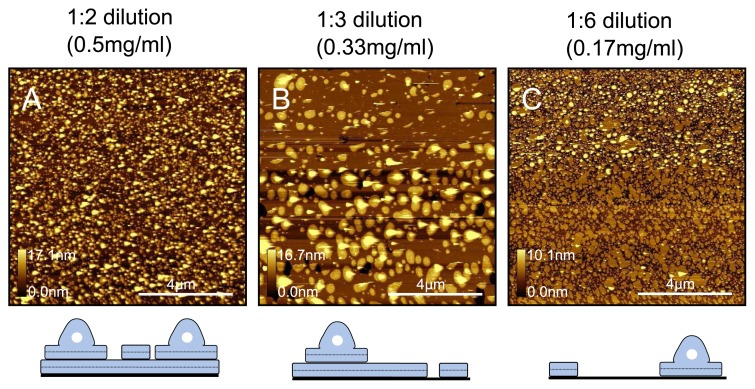
Dilution test for samples prepared at 33 °C. DPPC samples in water were prepared at (**A**) 0.5mgml^−1^ ; (**B**) 0.33mgml^−1^ and (**C**) 0.17mgml^−1^ . By diluting the liposome solution to the point where bare mica is seen in the samples, we are able to see the very initial stages of vesicle fusion, which indicate protrusions are partially fused vesicles. Below: Schematics illustrating proposed generalised models for respective samples.

Although we mentioned earlier that the protrusions are likely to be trapped vesicles, it is actually very difficult to be certain about the exact form that they take. As shown in Figure 7 we suggest that they could be either trapped vesicles, adsorbed vesicles or partially fused vesicles. In order to gain insights into this process, we prepared a new liposome solution at 1mgml^−1^ and diluted it to create samples at 0.5mgml^−1^, 0.33mgml^−1^ and 0.17mgml^−1^ and deposited at 33 °C. Higher resolution images of these samples are shown in Figure 8. At 0.5mgml^−1^, we observe a complete bilayer, but with partially fused bilayer patches adsorbed on top. When the solution is diluted to 0.33mgml^−1^, we see a decrease in the quantity of the adsorbed bilayer patches, and some holes in the first bilayer that expose the mica surface. When the solution is diluted to 0.17mgml^−1^, we see the mica is only partially covered, and that although there are some flat bilayer patches, a good proportion of them appear to exist in the partially fused state. It seems that preparing bilayers at these lower temperatures in water results in partially fused vesicles that lead to the protrusions observed. Although lower domains and holes can be eliminated at lower temperatures, the drawback is that partially fused vesicles are inevitable. Such a surface is not ideally suited as a model test surface. Samples created at higher temperatures, although lacking vesicle type protrusions, are also not ideal as the tip can more easily get contaminated at the edge of holes in the bilayer.

**Figure 9:**
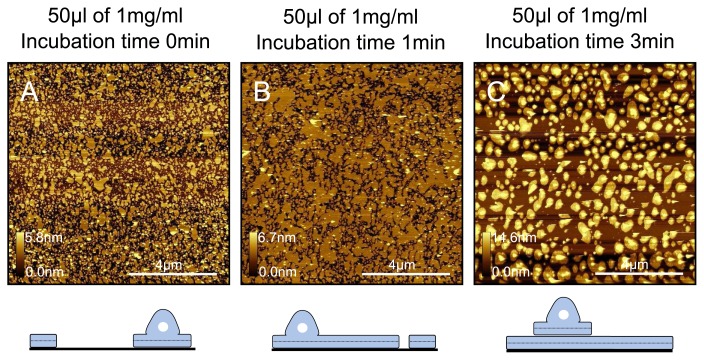
Variation of short incubation times when using a DPPC solution of high concentration (1mgml^−1^ ). Incubation times of (**A**) 0min; (**B**) 1min and (**C**) 3min. The increase in surface coverage with increasing incubation time is qualitatively the same as seen for experiments increasing the concentration.

Interestingly, we observe a very similar trend when varying the incubation time of a 1mgml^−1^ DPPC solution. As shown in Figure 9, we vary the incubation time from 0min (sample immediately washed after deposition, actual incubation time < 2 sec) to 3min. At the shortest incubation time, we observe patchy coverage across the mica surface, including partially fused vesicles. The coverage increases in a time-dependent manner, and at the longest incubation time we again observe a complete bilayer but with lipid patches and partially fused vesicles across the surface. Based on this, it seems that an increase in incubation time is equivalent to an increase in concentration. Another important point to note is that in this case these samples were prepared at room temperature. It seems therefore that although vesicles are not able to completely fuse when at lower concentrations (~ 0.3mgml^−1^ ) and below 33 °C, vesicles will completely fuse even at room temperature when the concentration is high enough. Therefore, this suggests that there is interdependence between the concentration and the temperature at which vesicles will fuse.

**Figure 10:**
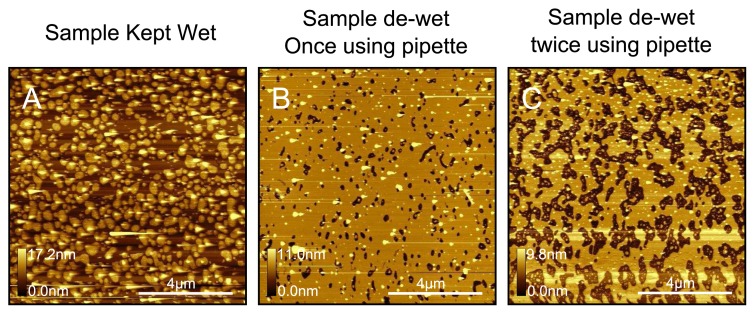
Illustration of the dewetting test for DPPC. The sample bilayer in (**A**), which is very similar to the bilayer in Figure 9C, was dewetted instantaneously and immediately rehydrated (**B**); The dewetting step was repeated a second time (**C**). The holes in the bilayer become progressively larger after each successive wash. The test proves that the sample in (**A**) was a complete bilayer with partially fused lipid patches on top.

In Figure 10 we demonstrate the dewetting test for DPPC. In this case we removed the liquid from the fluid cell carefully until the surface was instantaneously dewetted, after which water was immediately replenished. The surface was re-imaged, and then the dewetting step repeated again followed by a final image being taken. As can be seen, holes are formed after the initial dewetting, which become larger after the second dewetting step. As with DOPC, this is a good test used to verify that the surface was completely covered with a first layer of lipid. In addition we can see that dewetting seems to preferentially remove the bilayer patches from the top as opposed to the vesicle structures. As with DOPC, we also tested the effect of various washes with increased force but again observed no change in the bilayer topography. We also tested the effect of washing with a buffer at a different ionic strength (PBS) followed by again washing with water, for which we also observe no change in the surface topography.

### 2.3. DPPC Bilayers from Vesicles in Buffered Salt Solutions

We see in the literature that bilayers can be prepared both in pure water and in solutions containing buffers and salts of various types and concentrations. It is also reported in the literature that divalent cations such as Ca^2+^ or Mg^2+^ are very important in aiding fusion of vesicles to mica surfaces [42,43,51]. We therefore tested two buffers, one containing only NaCl and HEPES buffer (HEPES-NaCl: 10mM HEPES, 150mM NaCl, pH 7.4), and another in addition containing 20mM Mg^2+^ (HEPES-NaCl-Mg:10mM HEPES, 150mM NaCl, 20mM MgCl_2_, pH 7.4).

**Figure 11:**
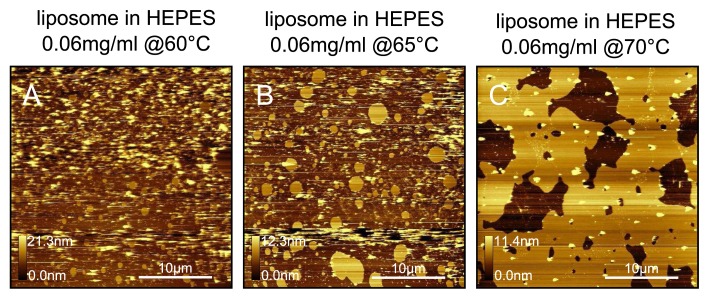
DPPC bilayers prepared from a HEPES-NaCl liposome solution. Bilayers prepared at (**A**) 60 °C; (**B**) 65 °C and (**C**) 70 °C. We observe continuous fusion only when the sample plate is maintained at 70 °C; below this temperature mostly observed are vesicles with occasional fused patches.

We see in Figure 11 that vesicles do not fuse to mica unless the deposition temperature is raised to 70 °C. Below this temperature, we see a combination of small fused bilayer patches and unfused vesicles.

**Figure 12:**
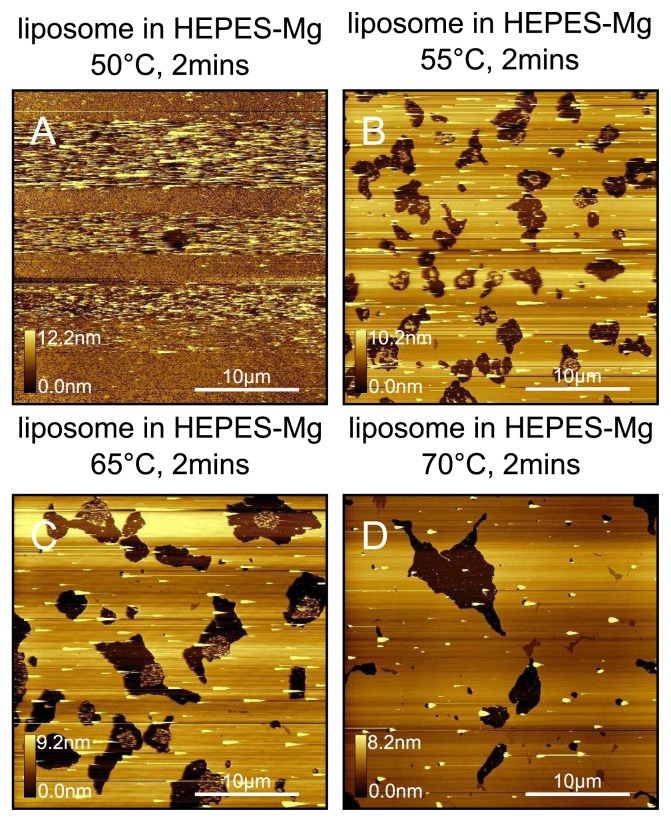
DPPC bilayers prepared from a HEPES-NaCl-Mg liposome solution. Bilayers prepared at (**A**) 50 °C; (**B**) 55 °C; (**C**) 65 °C; (**D**) 70 °C. When the sample plate is maintained at 50 °C we only observe a vesicle layer, which can be partially fused by the tip as indicated by the horizontal streaks in A. For 55 °C, 65 °C and 70 °C deposition temperatures, we observe extended regions of fused bilayer. More domains are observed for the highest temperatures. Samples were prepared using liposomes in HEPES-Mg at exactly the same concentration (0.06mgml^−1^ ) and deposition time (2min) as for the HEPES-NaCl samples shown in Figure 11.

Samples were prepared using liposomes in HEPES-Mg at exactly the same concentration (0.06mgml^−1^ ) and deposition time (2min) as for the HEPES-NaCl samples shown in Figure 11. We see from Figure 12 that at 50 °C a vesicle layer is formed. By increasing the force whilst scanning, we are able to fuse the vesicles to some extent as shown by the contrasting horizontal regions of fused and unfused regions. From 55 °C to 70 °C we clearly see that the bilayer has fused, however quite a lot of holes still appear. The change in minimum temperature for complete vesicle fusion of DPPC liposomes at 0.06mgml^−1^ between HEPES-NaCl buffer and HEPES-NaCl-Mg buffer from ~ 70 °C to ~ 55 °C is thought to be due to the increased attraction between positively charged liposomes and negatively charged mica. It has been shown that for DMPC liposomes, which have the same head group as DPPC, the charge on the liposomes can vary depending on the ionic strength of the medium [48]. In the case of pure water, they observe a strong repulsion due to negative charges, whereas in 100–150mM NaCl the liposomes are close to neutral. With 150mM NaCl plus 20mM MgCl_2_, they observe a repulsion due to positive charges.

**Figure 13:**
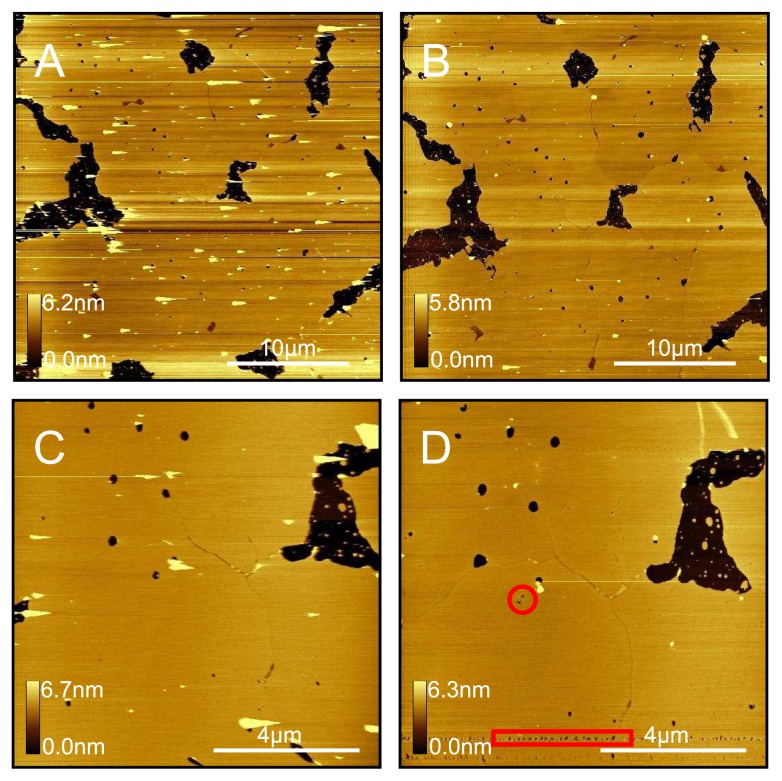
DPPC sample prepared from HEPES-NaCl-Mg liposome solution and incubated at 60 °C for 60min. (**A**) Light force scanning highlights vesicles; (**B**) Higher force scanning makes the sample appear more continuous than it really is. Although some vesicles were dislodged and swept away, the smoother appearance of the surface is mostly due to the tip tracking across the vesicles better; (**C**) Extended regions of defect-free bilayer are observed; (**D**) A few lines of (**C**) were scanned with a high force and then scanned lightly again (area enclosed by red box). We see holes due to the hard scanning. We also see holes disappearing even with relatively light scanning (area enclosed by red circle), indicating that the DPPC bilayers are delicate and dynamic, although much less so than DOPC.

Figure 13A shows another DPPC sample prepared in HEPES-Mg, similar to the samples shown in Figures 12B–D except that it was incubated at 60 °C and for 60min as opposed to just 2min. The sample is very similar to those prepared for shorter incubation times, indicating that increasing the time further has little effect on the quality of the bilayer. We note that by increasing the force that the tip images with, we are able to superficially make the sample appear smoother, with less trapped vesicles. Although some of the vesicles were dislodged and moved away whilst scanning, many of them still remain and the surface mainly looks much cleaner because the tip is tracking across the surface of the vesicles better with the higher force. At these higher forces however, damage can occur to the sample. We see a higher resolution scan in Figure 13C. A few lines of this area were then scanned with much increased force (Figure 13D), which then causes holes to appear with the second pass at a much lighter force. In addition to the formation of holes, we see that some holes fill in even when scanning relatively lightly. Although the extent of tip induced changes is far less than with fluidic DOPC, we still see that DPPC is quite easily deformed by the tip. We believe that the quality of the bilayer shown in Figure 13D is comparable to DPPC bilayers presented by several other groups, which used their DPPC samples as test systems to assess changes due to other proteins/peptides or drug interactions [16,23,25,30,47,50,52].

Overall we do not observe any significant improvement in the quality of the DPPC bilayer surface (extent of flat regions, absence of holes, domains or trapped vesicles) when comparing bilayers prepared in water (Figure 5C), HEPES-NaCl (Figure 11C) or HEPES-NaCl-Mg (Figure 12D). However, these samples were prepared with slightly different experimental conditions; pure water: *C_l_* = 0.3mgml^−1^, *t_d_* = 5min, *T_d_* = 60 °C, *R_c_* = 0.5 °Cmin^−1^ ; HEPES-NaCl: *C_l_* = 0.06mgml^−1^, *t_d_* = 2min, *T_d_* = 70 °C, *R_c_* = 5 °Cmin^−1^ ; HEPES-NaCl-Mg: *C_l_* = 0.06mgml^−1^, *t_d_* = 2min, *T_d_* = 55 °C, *R_c_* = 5 °Cmin^−1^ (*C_l_*, *t_d_*, *T_d_*, *R_c_* are lipid concentration, deposition time, deposition temperature and cooling rate respectively).

Generally we observe that DPPC bilayers prepared in either HEPES-NaCl or HEPES-NaCl-Mg form fused bilayers with less concentrated solutions and with less time than compared with vesicles prepared in pure water alone. In order to prepare fluid phase DOPC bilayers in pure water, we need higher concentration (*>* 0.5mgml^−1^ ) and longer incubation time (~ 15min). Generally we are able to use much faster cooling rates for DPPC samples prepared in either buffer and see fewer domains compared with samples prepared in pure water, for which a high proportion of the sample surface contains lower domains unless very slow cooling rates are used. Since DOPC is in the fluid phase at room temperature, we never observe domain formation during sample preparation. We also observe interdependence between lipid concentration and the minimum temperature at which vesicles fuse for DPPC. We observe that although vesicles are not able to fuse when at lower concentrations (~ 0.3mgml^−1^ ) and below 33 °C, vesicles will fuse even at room temperature when the concentration is high enough (1mgml^−1^ ).

### 2.4. Force Spectroscopy of DOPC Bilayers

**Figure 14:**
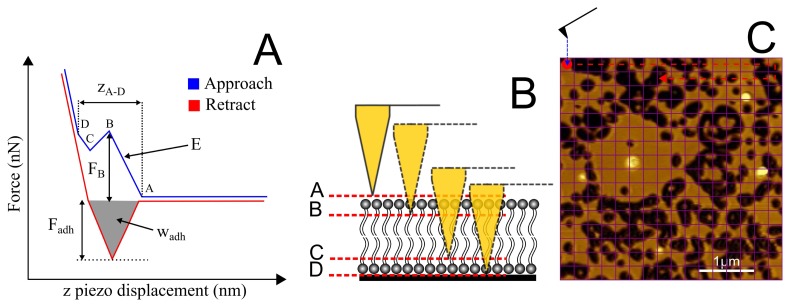
(**A**) Schematic force-distance plot highlighting the most significant features of a typical rupture event associated with a fluid-like lipid bilayer. From the approach curve (blue), the rupture force *F*_B_, the bilayer depth *z*_A–D_ and the Young’s modulus *E* may be determined. From the retract curve (red), the maximum force of adhesion *F*_adh_ and the work of adhesion *W*_adh_ may be determined; (**B**) Schematic illustrating tip penetration through the bilayer (not to scale); (**C**) A 16 × 16 grid illustrating “force volume mapping”, where force-distance curves are conducted at each point on a grid across a surface (in this case 4 *μ*m × 4 *μ*m).

During a typical force spectroscopy experiment, the tip successively approaches and leaves the surface in a cyclic manner. The force experienced by the cantilever is detected and then plotted against the z-piezo displacement or tip-sample separation. During each cycle the x- and y-coordinates are typically fixed, however in order to get a more reliable measure of the surface properties, the tip is typically moved laterally between cycles, in an approach often referred to as “force volume mapping” [53]. This is illustrated schematically in Figure 14C. Collecting data at several different points across a surface is preferable over measurements at a single point, since slight deviations in bilayer properties can be averaged, giving a more representative measurement. However, as seen in the image, if the bilayer is full of holes, the tip can easily become contaminated at the mica–lipid edges, resulting in misleading results. It is imperative therefore that the bilayer be continuous for such measurements.

Figure 14 illustrates the most important features of a typical force-distance curve exhibiting a rupture event associated with a fluid-like lipid bilayer. Typically, the rupture force (*F*_B_), bilayer depth (*z*_A–B_) and Young’s modulus (*E*) may be determined from the approach curve. In addition, the force of adhesion (*F*_adh_) and work of adhesion (*W*_adh_) may be determined from the retract curve. For the current work however, we focus just on the rupture force and depthmeasurements. All of the significant discontinuities in the approach curve have been labeled (A–D). The physical significance of these points and their transitions is interpreted as follows; (A) First contact of the tip with the top surface of the bilayer; (A–B) elastic compression of the bilayer; (B) Rupture of the upper surface of the bilayer; (B–C) Rapid tip transition through the central portion of the bilayer; (C) On-set of increased repulsion associated with compression of proximal head groups, water layer [54] and other trapped material; (C–D) Compression of trapped material; (D) Tip in direct contact with mica surface.

We are able to get several different measures of the bilayer depth. The full depth of the bilayer may be considered to be the distance between points A and D (*z*_B–D_). It should be noted however that several factors can lead to over- or under-estimation of the bilayer depth using this approach. Point A is not always well-defined, for example, due to long range electrostatic repulsion leading to a curved region around A, and a similar problem may be observed around D. The nature of the curve about these discontinuities is dependent on the tip chemistry, the ionic strength of the medium and lipid composition and phase. Typically ill-defined contact regions have been observed when a bilayer has formed on the tip, creating strong repulsion for example due to hydration effects [37,55]. For these reasons, the depth *z*_B–D_ is typically quoted in the literature and often referred to as the “jump depth”.

**Figure 15:**
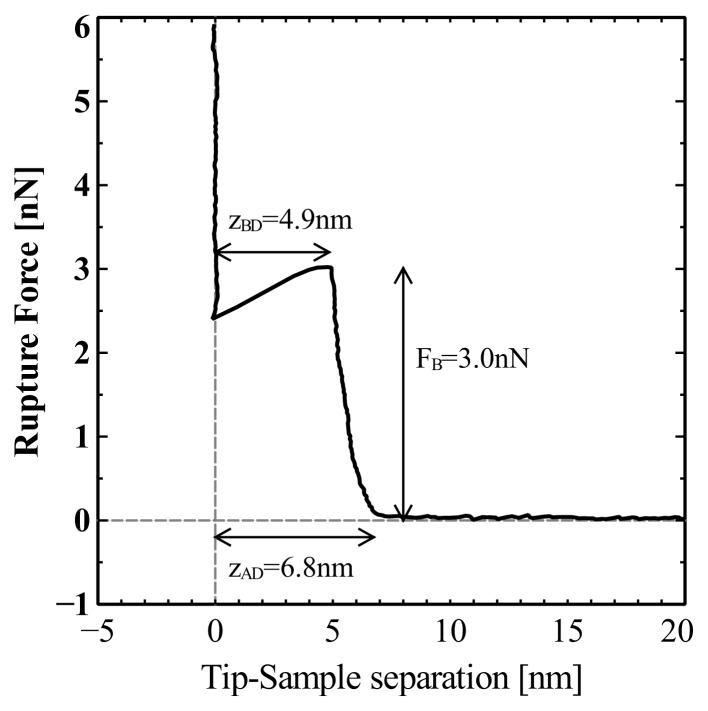
A representative force–distance curve for the rupture of DOPC in water. The rupture occurs at *F*_B_ = 3.0 nN. The tip first senses the bilayer at a distance *z*_A–D_ = 6.8 nm from the mica surface. The rupture depth occurs at *z*_B–D_ = 4.9 nm. The bilayer depth for DOPC is in good agreement with values reported by X-ray diffraction [54].

In Figure15 we show a single representative force distance curve for the rupture of DOPC in pure water. We have converted the z-piezo displacement to tip-sample separation so as to more accurately reflect the tip dynamics. We observe that the tip first senses the upper surface of the bilayer at a distance *z*_A–D_ = 6.8 nm from the mica surface and the rupture depth occurs at *z*_B–D_ = 4.9 nm. The rupture occurs at *F*_B_ = 3.0 nN.

**Figure 16:**
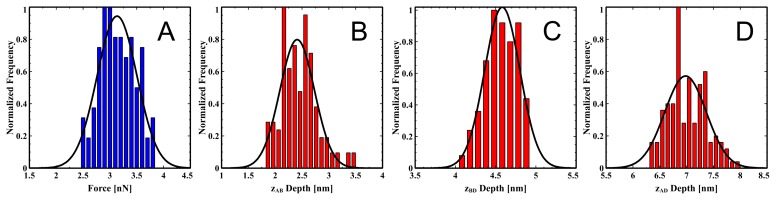
DOPC rupture force and depth distributions from a representative data set. (**A**) Rupture force, *F*_B_; (**B**) *z*_A–B_ depth; (**C**) *z*_B–D_ depth; (**D**) *z*_A–D_ depth. Mean values are *F*_B_ = 3.1 ± 0.3 nN, *z*_A–B_ = 2.4 ± 0.3 nm, *z*_B–D_ = 4.6 ± 0.2 nm, *z*_A–D_ = 7.0 ± 0.3 nm (errors are standard deviations, number of data points per distribution is 136). Black lines are Gaussian fits to the distributions.

In order to have a more accurate reflection of the bilayer properties, it is necessary to repeat the force-distance measurements several times. As shown in Figure 16 we have collected data for 136 approach–retract cycles for a single tip. The mean values are *F*_B_ = 3.1 ± 0.3 nN, *z*_A–B_ = 2.4 ± 0.3 nm, *z*_B–D_ = 4.6 ± 0.2 nm, *z*_A–D_ = 7.0 ± 0.3 nm.

The rupture force for DOPC is comparable to other reported values [27,34]. The *z*_B–D_ depth is in good agreement with the value of 5.0 nm reported here from the earlier image analysis of bilayer patches and agrees with other reports in the literature using the same approach where values of 5.5 nm [47] and 4.1 ± 0.2 nm were observed [56]. As mentioned before however, determining bilayer depths for soft DOPC samples using the imaging approach is typically unreliable due to the deformation that the tip causes and the strong susceptibility to small changes in the setpoint force, which is confounded by tip contamination by the soft lipid molecules. We have seen depth values ranging from ~ 3 nm to ~ 7 nm for DOPC using standard image analysis of DOPC patches on mica.

It is difficult to assign a “true depth” to the bilayer because that depends on from where depth is measured for these measurements. The value *z*_A–D_ = 7.0±0.3 nm from the force spectroscopy analysis is undoubtedly an overestimate, since the tip may sense electrostatic interactions before it actually makes contact, and the term “contact” is also ill-defined because the head groups are typically hydrated, so the tip may be first sensing those water molecules. The depth *z*_B–D_ = 4.6 ± 0.2 nm is taken at a point when the bilayer is in compression, and so is likely an underestimation. Interestingly, an X-ray diffraction study [54] reports a fully hydrated DOPC bilayer thickness of *D* = 6.31 nm, which is most similar to the depth *z*_A–D_ = 7.0 ± 0.3 nm. They observe the thickness only due to the lipids as being *D*^′^*_B_* = 4.53 nm, which is comparable to the depth *z*_B–D_ = 4.6±0.2 nm. They also report a water layer of *D*^′^*_W_* = 1.79 nm and comparable to the depth *z*_A–B_ = 2.4 ± 0.3 nm. We believe that presenting the constituent depth contributions, especially *z*_A–D_, *z*_B–D_ and *z*_A–D_, is a much more robust way of characterising a bilayer as compared with the standard image analysis approach.

## 3. Experimental Section

### 3.1. Materials and Instrumentation

Stock ampules (25mg) of 1,2-dioleoyl-sn-glycero-3-phosphocholine (DOPC, purity *>* 99%) in chloroform were purchased from Avanti Lipids and stored at −20 °C immediately after receipt. Powdered 1,2-dipalmitoyl-sn-glycero-3-phosphocholine (DPPC, purity ≥ 99%) was purchased from Sigma-Aldrich and stored at −20 °C. Chloroform (purity *>* 99.8%) and sodium chloride (purity *>* 99%) were purchased from EMD chemicals (USA). Methanol (purity *>* 99.8%), sodium hydroxide (purity *>* 97%) and magnesium chloride (purity *>* 99%) were purchased from Caledon Laboratories (Georgetown, Ontario, Canada). HEPES (purity *>* 99.5%) was purchased from Sigma-Aldrich. Millipore water (resistivity *>* 18Mcm) from a Synergy UV-system was used throughout. A Branson 1510 sonicator bath was also used. Muscovite mica (grade V-4, 22mm diameter, 0.15mm thick circular discs) was purchased from SPI Supplies (West Chester, PA, USA). Silicon MAC-2 cantilevers (nominal spring constant 2.8Nm^−1^ ) were purchased from Agilent Technologies. DNPS silicon nitride cantilevers (4 levers, nominal spring constants range in 0.06Nm^−1^ – 0.350Nm^−1^ ) and gold coated NPG levers (4 levers, nominal spring constants range in 0.06Nm^−1^ – 0.350Nm^−1^ ) were purchased from Bruker. 1-Undecanethiol (purity *>* 98%) was purchased from sigma.

### 3.2. General Handling of Lipids

An important point to note when handling DOPC is that due to the double bond in its tail, it is particularly susceptible to hydrolysis or oxidation [57]. The lipid powders are extremely hygroscopic and so we prefer to purchase DOPC dissolved in chloroform and layered with argon. Upon receipt, we store at −20 °C and use one ampoule at a time, which can be divided into aliquots, layered with nitrogen/argon and again stored at −20 °C until required. Degassing water with nitrogen/argon to displace oxygen before liposome preparation and always keeping solutions of DOPC layered with nitrogen/argon when not in use are also recommended.

### 3.3. DOPC Bilayer Preparation

#### Stock Solution Preparation

1. Open the ampule containing 25mg DOPC in chloroform.
2. Transfer the entire ampule directly to a 20ml glass vial. Add an extra 1000 *μ*l of chloroform to the ampule and wash out to the glass vial so as to remove all lipid.
3. Evaporate the solvents under a stream of nitrogen until visibly dry (approximately 10–15 min).
4. Add 6ml chloroform/methanol (2:1) and split into even aliquots to 6 small glass vials (capacity
5. mL each). Each stock vial therefore contains 4.17mg DOPC.
6. Top with nitrogen and store at −20 °C.

#### Liposome Preparation

1. Transfer one of the 1000 *μ*l stock aliquots to a clean 20ml glass vial. Wash out the small vial with an extra 1600 *μ*l chloroform/methanol (2:1) and add this to the 20ml vial as well to make 2mM and ensure no lipid is lost.
2. Evaporate solvents with a continuous stream of nitrogen. After ~ 15min all should be visibly dry. Continue for an extra 15min to ensure all solvents are evaporated (30min total).
3. Bubble nitrogen gas through ~ 20ml of millipore water for ~ 15min to remove the oxygen.
4. Add de-oxygenated millipore water to make 0.5mgml^−1^ . The lipid should quickly start to swell, separate from the glass vial and form an inhomogeneous suspension of cloudy material. Also add a stir bar and top the vial with nitrogen.
5. Stir using magnetic stirrer for 30min at 1100 rpm (the solution should appear homogeneous and milky).
6. Place the solution at 4 °C and allow to swell for 1 hour.
7. Stir using magnetic stirrer at 1100 rpm for 30min at room temperature.
8. Place the vial in the middle of the sonicator where cavitation is greatest. The most powerful region of any bath sonicator can be found easily by placing a sheet of aluminium foil on the surface of the water and sonicating for ~ 10min—a large hole should appear at the region of most intense power. Sonicate for 30min, during which we observe slight heating of the water (start temperature is ~ 23 °C, finish temperature is ~ 31 °C. The solution should appear completely clear after sonication. If using higher concentrations of lipid, the solution may take longer to go to clarity.
9. Store the liposome solution at 4 °C until needed.

#### Vesicle Fusion

1. Remove the liposome solution from the fridge and stir at 1100 rpm for ~ 45 s.
2. Place 300 *μ*l of cold solution directly into the AFM fluid cell containing freshly cleaved mica.
3. Wait 15min.
4. Wash with 10ml water through a syringe by slowly allowing the fluid cell to overflow so as to prevent sample de-wetting.

#### Optimization

Typically the above protocol will give a continuous bilayer of DOPC. However, slight decreases in the concentration (e.g., a little extra lipid lost during preparation of the liposome solution) may mean that a complete bilayer is not formed. In this case we have found that it is best to prepare several samples that have been incubated for slightly longer and shorter periods of time and image these using the AFM. By capturing the state of bilayer coverage at various points in time, it is then possible to be certain that for a given incubation time a complete bilayer was formed. The time series test is described in more detail in the main text. In some cases, a complete bilayer may not form even for extended incubation times. In this case, another approach can be taken whereby a higher concentration of lipid (1mgml^−1^ ) liposome solution is prepared. This solution can then be sequentially diluted to find the optimum concentration to form a complete bilayer.

### 3.4. DPPC Bilayer Preparation

#### Liposome Preparation

1. Weigh out powdered DPPC and add chloroform/methanol (2:1) to make 2mM.
2. Evaporate solvents with a continuous stream of nitrogen for ~ 30min or until visibly dry.
3. For liposomes in water: Add water to make 0.3mgml^−1^ (this concentration was used for the sample shown in Figure 5). For liposome in buffer: Add HEPES buffer (10mM HEPES, 150mM NaCl, 20mM MgCl_2_, pH 7.4) to make 1mgml^−1^ (this liposome concentration was used for samples shown in Figure 13 but diluted later). Also add a stir bar and top the vial with nitrogen.
4. Stir using magnetic stirrer for 30min at 1100 rpm and at room temperature.
5. Place the solution at 60 °C and allow to swell for 1 hour.
6. Stir using magnetic stirrer for 30min at 1100 rpm.
7. Sonicate for 45min at 60 °C. The solution should appear completely clear after sonication. However, if using higher concentrations of lipid, the solution may take longer to go to clarity.

#### Vesicle Fusion

1. Stir the liposome solution for ~ 45 s.
2. Prepare the fluid cell with freshly cleaved mica at 60 °C.
3. For liposomes in water: Add 300 *μ*l and wait 5min. For liposomes in buffer: dilute to ~ 0.06mgml^−1^ in a small centrifuge tube, add 300 *μ*l to the fluid cell and wait 2min.
4. For liposomes in water: Cool at a rate less than 0.5 °Cmin^−1^ . For liposomes in buffer: Cooling at 5 °Cmin^−1^ will produce a few domains, and a lower cooling rate will produce even fewer.
5. Wash with 10ml water through a syringe by slowly allowing the fluid cell to overflow so as to prevent sample de-wetting.

### 3.5. Atomic Force Microscopy

All AFM imaging was conducted in pure water or buffer either using an Agilent 5500 AFM (Agilent technologies Inc, Santa Clara CA, USA) equipped with a MAC mode 3 control box, or a JPK nanowizard-2 (JPK Instruments AG, Berlin, Germany). Temperature controlled experiments were performed using the Agilent system equipped with a LakeShore 325 temperature controller and fluid cell. Imaging was performed in intermittent contact mode or MAC mode (Agilent AFM only) using MAC-II cantilevers (nominal *k* = 2.8Nm^−1^ ). MAC mode allows for excellent control of the cantilever in liquid environments and is ideally suited for imaging soft supported bilayer surfaces. Since the mode is an intermittent contact (or AC) technique, cantilevers of slightly higher spring constant than those used for contact mode can be used with great effect. We have also imaged in contact mode using DNPS (Bruker) levers with a nominal spring constant of 0.06–0.350Nm^−1^ and find the image quality comparable. However, MAC mode is less sensitive to cantilever drift (as the cantilever deflection drifts the force set-point must be continually adjusted to maintain a constant contact force) and so is often more convenient.

### 3.6. Atomic Force Spectroscopy

For force spectroscopy experiments presented here, we used gold coated probes from Bruker (NPG, nominal *k* = 0.06Nm^−1^ ). Cantilevers were first functionalised with *>* 2mM 1-undecanethiol for *>* 12 h to provide a more homogeneous surface. As with Loi *et al*. [58] we observe improved consistency with this approach rather than using uncoated levers. Several different samples and cantilevers have been tested, all showing qualitatively the same results. We have also replicated experiments using uncoated MAC-2 silicon cantilevers from Agilent.

Cantilevers were first calibrated in air using the thermal tune method [59], which is implemented in both the Agilent PicoView software and the JPK software. Briefly, a thermal noise spectrum is first recorded in air. The cantilever is then pressed against a hard material such as mica to obtain the cantilever sensitivity in mV^−1^ . The cantilever spring constant is then calculated from these two measurements as detailed in the reference.

Force curves were obtained by repeatedly approaching and retracting the cantilever to the surface (tip velocity was 715 nms^−1^ ) whilst simultaneously recording the tip deflection. The tip deflection data *V_d_* (in Volts) was converted to force units (Newtons) using *F* = *kV_d_S*, where *S* is the normal cantilever sensitivity in mV^−1^ . The normal sensitivity is the inverse of the gradient of the linear region of the force-distance curve when the cantilever is in hard contact. The force calibration was carried out for each force curve separately to ensure the most accurate value for the sensitivity in case of drift. Care was taken to ensure that high enough loads were reached such that the sensitivity could be found from a linear region. The z-piezo displacement values, *z*_d_, were converted to tip-sample separations, *z*_ts_, using *z*_ts_ = |*z*_p_| – |*z*_d_|, where *z*_d_ is the tip deflection in nanometers. Typically force curves are taken at points on a grid of 16 by 16 points across a 2 *μ*m square area. Several different areas have also been tested and give qualitatively the same results. Results were analysed using a script developed in our laboratory with MATLAB 7.4.0.

## 4. Conclusions

We have developed robust protocols to reproducibly generate extended regions of DOPC bilayer surfaces on mica in water using the vesicle fusion approach. Such test surfaces are typically defect-free and thus ideally suited for studying the influence of effector molecules such as proteins/peptides or drugs either through AFM imaging or by force spectroscopy. Such surfaces are especially suitable for force spectroscopy studies, in which the tip can easily get contaminated at edges of defects in the bilayer. We also demonstrate that DPPC bilayers can be prepared either in pure water or in buffer solutions with additional ions. These surfaces typically have defects either in the form of lower domains or holes exposing bare mica. However, such defects are reported by several other groups and there are still regions ~ 5 *μ*m^2^ that are suitable for further testing.

The large number of different experimental parameters that can influence both the preparation of the liposome solution and their fusion with mica, together with the interdependence of some of those parameters, make it very difficult to understand and control the whole process. There are a staggering number of laboratories that have their own unique “recipe” for preparing bilayers on surfaces, and the complexity of bilayer preparation demonstrated here undoubtedly contributes to such diversity. We have focused here on just two examples of lipids containing phosphocholine (PC) head groups in their fluid and gel phases. It is expected that different lipids will require different conditions in order to consistently prepare bilayers suitable as model systems, however the general themes should apply to all. We hope that by highlighting the most critical experimental parameters, and by providing their general protocols, we provide crucial information to aid scientists, especially those new to the field.

We have also demonstrated force spectroscopy studies of DOPC bilayers prepared using our vesicle fusion protocol. Both the depth measurements and rupture force data are comparable to literature values. We also demonstrate an approach for the assessment of the bilayer thickness characteristics. We sub-divide the force-distance plots into depths about the discontinuities and find that these compare well to hydrated and unhydrated lipid values obtained by X-ray diffraction. Without a robust and reliable protocol for bilayer preparation, much time can be wasted with inconsistent and misleading results. With the protocol that we present here however, force spectroscopy experiments can be quickly used to assess lipid surfaces in a reliable manner.
